# The effect of the time parents spend with children on children's well-being

**DOI:** 10.3389/fpsyg.2023.1096128

**Published:** 2023-04-03

**Authors:** Dongxu Li, Xi Guo

**Affiliations:** ^1^School of Public Administration, Inner Mongolia University, Hohhot, China; ^2^Inner Mongolia University of Technology, Hohhot, China

**Keywords:** time with children, well-being, time use survey, the role of parents, mental health

## Abstract

**Background:**

The time spent with parents is a crucial factor in the growth of children, and children's well-being is an important indicator of their mental health.

**Methods:**

To promote the children's well-being, this study, which is relying on the data from the 2017 China Time Use Survey (CTUS), explores the relationship between parental time and children's well-being and specific influencing factors.

**Results:**

The more time parents spent with children, the higher their children's well-being will be (coefficient 0.1020, *p* < 0.01). The life and leisure time parents spent with children promoted children's well-being (coefficient 0.1020, *p* < 0.01). The life and leisure time the mother spent with children (coefficient 0.1030, *p* < 0.05) the life and leisure time (coefficient 0.1790, *p* < 0.05) and the educational interactions time the father spent with children (coefficient 0.3630, *p* < 0.10) positively affected children's well-being. The influence of the time parents spent with children on children's well-being was heterogeneous based on their children's academic performance.

**Conclusions:**

Parental accompaniment is an important determinant of children's well-being. Family education, guidance services, and mental health services should be strengthened, and it is necessary to improve the time spent with children and to pay attention to individual differences in children.

## 1. Introduction

Children conceptualize well-being as positive experiences in their life (Vujcic et al., 2019). Many studies have demonstrated the cognitive ability of children to think meaningfully about their subjective well-being; positive and negative effects are key components of children's well-being (Savahl et al., 2021). Parents remain one of the important factors influencing children's well-being (Rees and Dinisman, 2015; Fallesen and Ghler, 2019). Parents are the main participants in children's lives, and the main goal for parents is to establish an active caregiver role among their children (Rafferty et al., 2020). In the parent–child relationship, parents' emotional warmth can not only provide a positive emotional atmosphere to the child but also play a positive role in the psychological regulation of their child (Liu et al., 2022). Children need parents' care, seek unconditional acceptance, and recognition (Thoilliez, 2011); parents need to ensure an active and healthy life, and an optimistic future for children (Park et al., 2022). In families, parents invest financial and emotional resources to promote the well-being of their children (Mínguez, 2019). However, in practice, parents' contribution to the family is more focused on investing time in work to provide financial support to the family, with less attention to the impact of companionship and insufficient attention to children's emotional needs, which could result in barriers to growth for children. The companionship of parents brought closer connections between parents and children (Chi et al., 2019), and children also had the need for parental companionship and emotional interaction. This study, therefore, focused on the following issues: first, observing the effect of time spent with children on children's well-being from the perspective of parental time. Second, the study analyzed the difference in the amount of time children spend with fathers and mothers and the difference in the effect on children's well-being in terms of the division of parental roles. Finally, this study further analyzed whether the effect of time spent by parents with their children on the children's well-being differs according to children's academic performance.

### 1.1. The quality of parental companionship and the children's well-being

The need for group family activities and family experiences with parental involvement were a source of well-being for children (Maftei et al., 2020). Companionship with parents enabled children to express their thoughts and feelings freely and reduced children's negative attitudes (Jimenez et al., 2019); more frequent interaction with parents promotes well-being (Savahl et al., 2019; Nie et al., 2020). More time spent with children translated into higher children's well-being (Gugl and Welling, 2011). It was not only the frequency of time that mattered but also how the time was spent and the quality of the time. When the quality of time was not high, the quality of the parent–child relationship was lower (Roeters et al., 2010). The time spent with children should be high quality interaction, and more quality interactions were associated with greater success (Milkie et al., 2010). Children who were actively accompanied were more likely to have a high sense of well-being in life (McAuley et al., 2012; Carvalho et al., 2021).

The benefits to children were obvious when parents took the best care of them (Bonke and Greve, 2012). Children who had positive parent–child relationships and were sufficiently loved were likely to be happier (Güngör et al., 2019). The quality of time spent together significantly influenced parental childcare outcomes, and high-quality time spent together had a positive impact on all types of parenting (Kalenkoski and Foster, 2008). High-quality time focused on positive communication (Vaterlaus et al., 2019). Children with parents who were willing to create joy and emotionally warm companionship tend to develop a healthy well-adjusted personality with a high index of well-being (Fan et al., 2020). If a child had a poor relationship with their parents, the probability of physical problems would increase (Hagquist, 2016). When parents lack quality companionship, children's character development was difficult to achieve and sustain (Gahramanov et al., 2019). Therefore, we propose the following hypothesis H1:

H1: The longer the parental presence, the higher the children's well-being.

### 1.2. Parent–child relationship quality and its relationship with child adjustment

Most of the literature on the relationship between parent characteristics and performances and child adjustment has focused on the quality of that parent–child relationship. Parental socialization referred to the process by which parents transmitted their habits and values of the culture of origin so that the child could adopt an adequate functioning within the culture to which he or she belongs (Maccoby and Martin, 1983; Martinez-Escudero et al., 2020; Climent-Galarza et al., 2022). One of the first models that emerged about parental socialization was Baumrind (1968) Y model. This model proposed three parental styles: authoritative, authoritarian, and permissive (Baumrind, 1971), which corresponded to three modes of parental control, authoritative control, authoritarian control, and lack of control (i.e., permissive control) (Baumrind, 1968).

The model that has had the greatest impact and that has given rise to most of the research on parenting was the two-dimensional model of Maccoby and Martin (1983). According to this model, parents used two independent dimensions to socialize with their children and relate to them. These parenting dimensions were warmth and strictness (Maccoby and Martin, 1983; Lamborn et al., 1991; Darling and Steinberg, 1993; Garcia and Gracia, 2009; Martinez et al., 2020; Martínez et al., 2021; Queiroz et al., 2020; Climent-Galarza et al., 2022; Fuentes et al., 2022; Gimenez-Serrano et al., 2022; Palacios et al., 2022).

Parental warmth was the extent to which the parents showed the children love, approval, acceptance, and affection, and giving them their support (Gimenez-Serrano et al., 2022), establishing sensitivity, affection of parents when dealing with their children, the quality of the dialogue they engaged in Climent-Galarza et al. (2022), communication and reasoning with them (Martínez et al., 2019; Martinez et al., 2020), and communication using inductive reasoning (Darling and Steinberg, 1993; Palacios et al., 2022). Other labels used in the literature to refer to parental warmth were responsiveness, involvement, acceptance or implication (Darling and Steinberg, 1993; Garcia and Gracia, 2014; Martinez et al., 2020), or affection (Martinez-Escudero et al., 2020).

Parental strictness was the extent to which parents used discipline toward their children, controlled and/or supervised their behavior (Gimenez-Serrano et al., 2022), established norms for children's behavior, maintained a position of authority (Baumrind, 1991b; Darling and Steinberg, 1993), placed demands on children to promote compliance (i.e., the degree of imposition, authority, or rigidity) (Climent-Galarza et al., 2022), physical and/or verbal coercion, and demanding attitudes (Lamborn et al., 1991; Martínez et al., 2021; Palacios et al., 2022). Other labels used in the literature to refer to parental strictness were demandingness (Palacios et al., 2022), control, firmness (Darling and Steinberg, 1993; Steinberg, 2005), imposition (Martinez-Escudero et al., 2020), or supervision (Garcia et al., 2020). In summary, this may mean that different parental styles were accompanied by different companionship activities.

According to Maccoby and Martin's model, the two main parenting dimensions (i.e., warmth and strictness) combined and gave rise to four parental styles: authoritarian (strictness but not warmth), authoritative (strictness and warmth), indulgent (warmth but not strictness), and neglectful (neither strictness nor warmth) (Maccoby and Martin, 1983; Darling and Steinberg, 1993; Chen et al., 2016; Acar et al., 2017; Garcia et al., 2020; Perez-Gramaje et al., 2020; Queiroz et al., 2020; Villarejo et al., 2020; Climent-Galarza et al., 2022; Fuentes et al., 2022; Gimenez-Serrano et al., 2022; Palacios et al., 2022).

A vital aspect in the literature on parental socialization was which parental characteristics in their relationship with their children (parenting dimensions or parental style) were associated with better child adjustment in different cultural contexts. Classical studies conducted in Anglo–Saxon contexts with European–American samples (mostly white middle-class families) stated that the combination of parental warmth and parental strictness together (i.e., authoritative style) was related to the best child psychosocial adjustment (Baumrind, 1991a; Lamborn et al., 1991; Steinberg et al., 1991, 1994; Darling and Steinberg, 1993); however, this combination was not always associated with the best child psychosocial adjustment in all cultural contexts. Other studies conducted in ethnic minority groups in the United States, such as Chinese Americans (Chao, 2001) or African Americans (Deater-Deckard et al., 1996), and Arab societies (Dwairy and Achoui, 2006), stated that parental strictness without parental warmth (i.e., authoritarian style) was associated with the best child adjustment.

Contrary to classical studies, the most recent evidence conducted in European and Latin American countries supported the idea that parental warmth without parental strictness (i.e., indulgent style) was associated with the best child psychosocial adjustment, such as self-concept (Garcia et al., 2018, 2020; Garcia and Serra, 2019; Martinez-Escudero et al., 2020; Perez-Gramaje et al., 2020; Gimenez-Serrano et al., 2021, 2022; Fuentes et al., 2022; Palacios et al., 2022), empathy (Fuentes et al., 2022), satisfaction with life (Gimenez-Serrano et al., 2022), and psychosocial maturity (Garcia and Serra, 2019), which was a protective factor for both traditional bullying and cyberbullying victimization (Martínez et al., 2019). Children raised by warm but non-strict parents also show lower levels of child maladjustment, such as aggressiveness, emotional instability, behavioral problems (Garcia and Gracia, 2009; Garcia and Serra, 2019; Villarejo et al., 2020; Gimenez-Serrano et al., 2022), nervousness and hostile sexism (Gimenez-Serrano et al., 2022), and negative self-efficacy (Palacios et al., 2022). Similar to parenting style, the role of parents themselves could also have an impact on children's well-being. Parents spent different amounts of time depending on the type of activity the child was doing (Renk et al., 2003), with mothers spending more time with children (Cha and Song, 2016). This may mean that children became more accustomed to their mothers' care and therefore had a lower impact on well-being (Craig, 2006). Children were happier with their fathers, while mothers were more demanding of their children. Therefore, we propose hypothesis H2 as follows:

H2: The children's well-being is more influenced by the father's companionship than by the mother's companionship.

In summary, existing studies have focused on the impact of parenting on children's well-being. This provides a theoretical basis for examining the effect of parental companionship on children's well-being. Nevertheless, studies on the time parents spend with children and children's well-being left important research gaps. To further explore the factors of companionship that affect children's well-being, we addressed this issue from a time-use perspective. In addition, the roles of mothers and fathers differed in the formulation and implementation of norms (Vanassche et al., 2012). However, the existing literature was inadequate in discussing the delineation of parents' roles in spending time with their children. To address this gap in knowledge, by relying on China Time Use Survey (CTUS) data, this study used objective and specific data on parents' time spent with their children to show the relationship between the time with children and children's well-being. More specifically, we assessed (1) the relationship between the time parents spent with children and children's well-being, (2) whether there was a difference in the time and influence of the father's and mother's division, and (3) whether this difference was heterogeneous depending on the children's academic performance.

## 2. Data and methods

### 2.1. Data and participants

A time use survey could show the time spent by individuals in various activities over 24 h. These various activities cover all human activities and do not overlap with each other. From the perspective of time utilization, this approach could intuitively and reliably reflect an individual's response to a certain event and the degree of importance.

Two time use surveys were carried out in China. The first survey was conducted in 2008, and the 2017 CTUS was the most recent survey (Liang and Ji, 2020). The survey subjects were all family members at least 3 years of age from urban and rural sample households in 29 provinces in mainland China. The sample households were randomly selected from ~40,000 sample households in the 2017 China Household Finance Survey, including ~12,000 households. At the time of the survey, the interviewer was asked to interview each member of the sample household face-to-face whenever possible, and another member of the household could answer on his or her behalf when the respondent was younger than 12 years of age. Each respondent was required to complete a 24-h (4:00 a.m. the day before the survey to 4:00 a.m. the day of the survey) time log with a minimum time interval of 10 min (Du et al., 2019).

This study excluded related transportation activities and waiting time while parents engaged in activities with children. In categorizing the types of companionship, based on existing literature, questionnaire design, and research needs, activities such as taking care of children's life and leisure time together were defined as life and leisure time, and tutoring children with homework, studying with children, reading with children and related activities, waiting, and other educational activities were defined as educational interactive time. See Table 1 for the specific classification and explanation. This study chose a sample of typical 1-day responses, excluding the contingency of the time arrangement, and the subjects were between 7 and 16 years of age. To ensure that each father and mother has spent time with their child, this study included families of three with both parents, and the total number of matched children's samples was 515.

**Table 1:** Specific classification of parental companionship activities.

**Large category**	**Middle category**	**Subcategory**	**Description**
Parents' time spent with children	Life and leisure	Taking care of children's everyday needs	Refers to dressing, ordering stationery, feeding, bathing, medicine feeding, medical care, etc.
		Watching TV with children	Refers to activities that accompany minor family members to watch TV together.
		Playing with children	Refers to accompanying underage family members to playgrounds, hospitals, schools, and other places for related activities.
	Educational interaction	Tutoring children on their homework	Refers to activities that involve tutoring or supervising minor family members to complete their academic assignments.
		Studying with children	Refers to minor family members who accompany them when they are studying, such as urging their children to study.
		Reading with children	Refers to accompanying minor family members and involves reading books, newspapers, magazines, etc. (including paper and electronic media).
		Waiting activities related to caring for underage family members	Refers to the waiting activities that occur when picking up minor family members, such as waiting for the child when picking up the child at the school gate and returning home from school.
		Other activities to take care of minor family members	Refers to activities such as registering for the child, participating in various activities stipulated by the child's school, holding parent meetings, and accompanying the child to a museum.

### 2.2. Measures

The indicator for measuring well-being in this article comes from the respondent's answer to the survey question “In general, do you feel well-being now?” The questionnaire uses a 5-point Likert scale. Based on previous research (Du et al., 2020), we divided the responses into 5 categories, where 5 denoted excellent and 1 denoted poor.

The independent variable in this article was the time (in hours) parents spent with children, which referred to the total time parents spent on care activities for children in various aspects of life, study, etc.

The index used in this article to measure health was derived from the answer of “How was your current physical condition?” The indicator for measuring sleep quality in this article came from the answer to “How did you sleep last night?” The questionnaire used a 5-point Likert scale. According to the questionnaire setup, we divided the responses into 5 categories, in which 5 denoted excellent and 1 denoted poor. The index used in this article to measure academic performance was derived from the answer to “Your overall academic performance in this class was at?” The academic performance was set as a dichotomous variable, and those who answered “poor,” “below average” and “average” were the group with worse academic performance and were assigned a value of 0 and those who answered “above average” and “excellent” showed better academic performance and were assigned a value of 1. In summary, the control variables in this study included household income, academic performance, sleep quality, gender, age, urban and rural living status, ethnicity, and physical health, and they were used to provide personal and family information regarding demographic characteristics, resources, and time availability. The information for each variable is shown in Table 2.

**Table 2:** Identification and specific operations of various variables.

**Variable**	**Index**	**Measure**
Dependent variable	Well-being	Poor = 1, below-average = 2, average = 3, above-average = 4 excellent = 5
Independent variable	Parents' time spent with children	Refers to activities that involve accompanying children in all aspects of life and study.
Control variable	Age	7–16
	Gender	Male = 0, female = 1
	Area	Urban = 0, rural = 1
	Nation	Others = 0, The Han nationality = 1
	Family income	Total income of family members
	Physical condition	Poor = 1, below-average = 2, average = 3, above-average = 4, and excellent = 5
	Sleep quality	Poor = 1, below-average = 2, average = 3, above-average = 4, and excellent = 5
	Academic performance	Poor, below-average and average = 0, above-average and excellent = 1

### 2.3. Plan of analysis

Since the dependent variable is a multi-categorical variable, the independent variable was a combination of multiple factors that may affect the dependent variable, and the data type of most variables was mainly categorical data, showing a discrete state. Therefore, the ordered logistic model research method was more applicable.

## 3. Results

### 3.1. Sample characteristics

Participants' individual social characteristics are summarized in Table 3. Among the 515 participants, 221 of them (44%) were men and 294 (556%) were women; 474 (92%) out of 515 participants were the Han nationality and 41 (8%) were others; 421 (82%) out of 515 participants were living in an urban area and 94 (18%) were living in a rural area. The well-being per individual was 3.8932, the average time spent with children was 1.0509 h, the average family income was RMB¥ 118,344, the average physical condition was 4.1845, the average sleep quality was 4.2311, and the average academic performance score was 0.5728. See Table 3 for details.

**Table 3:** Descriptive statistics of each variable.

**Personal characteristics variables**	***N***	**Percentage**	**Other variables**	**Mean**	**Min**.	**Max**.
Men	221	44%	Well-Being	3.8932	1	5
Women	294	56%	Parents' time spend with children	1.0509	0	17.75
The Han nationality	474	92%	Family income	118,344	−158,624	5,000,000
Others	41	8%	Physical condition	4.1845	1	5
Urban	421	82%	Sleep quality	4.2311	1	5
Rural	94	18%	Academic performance	0.5728	0	1

### 3.2. Ordered logistic models

#### 3.2.1. Time spent with children and children's well-being

The correlation between the time parents spent with children and children's well-being and the marginal effect were tested with the ordered logistic model. The specific model estimation results are shown in Table 4. Table 4 shows the results of regressing the total time parents spent with children on children's well-being. The results indicated that the regression coefficient was >0, that was, the more time parents spent with children, the higher their children's well-being. The marginal effect indicated that, for every additional hour of companionship, the probability of below-average well-being was reduced by 0.21%, the probability of average well-being was reduced by 1.68%, the probability of above-average well-being was increased by 0.31%, and the probability of having excellent well-being was increased by 1.62%. In other words, as the time the parents accompanied increased, the well-being of children was transformed from poor changes to excellent changes. See Table 4 for details.

**Table 4:** Results of regressing the time parents spend with children on children's well-being.

	**Regression coefficients**	**Margins**				
		**Poor**	**Below-average**	**Average**	**Above average**	**Excellent**
Parents' time spent with children	0.1020^***^	−0.0004	−0.0021^**^	−0.0168^***^	0.0031^*^	0.0162^***^
	(2.88)	(−1.27)	(−2.19)	(−2.89)	(1.79)	(2.93)
Gender	−0.1520	0.0006	0.0031	0.0250	−0.0046	−0.0240
	(−0.87)	(0.79)	(0.87)	(0.87)	(−0.83)	(−0.87)
Age	−0.6920^**^	0.0027	0.0141^*^	0.1130^**^	−0.0210^*^	−0.1090^**^
	(−2.45)	(1.19)	(1.91)	(2.49)	(−1.68)	(−2.48)
Age^2^	0.0302^**^	−0.0001	−0.0006^*^	−0.0050^**^	0.0009^*^	0.0048^**^
	(2.52)	(−1.21)	(−1.95)	(−2.57)	(1.70)	(2.56)
Nation	0.1730	−0.0007	−0.0035	−0.0283	0.0052	0.0273
	(0.63)	(−0.58)	(−0.63)	(−0.63)	(0.62)	(0.63)
Areas	0.3620	−0.0014	−0.0074	−0.0593	0.0110	0.0572
	(1.53)	(−1.01)	(−1.42)	(−1.55)	(1.32)	(1.54)
Family income	0.0895^*^	−0.0003	−0.0018	−0.0147^*^	0.0027	0.0141^*^
	(1.91)	(−1.13)	(−1.56)	(−1.95)	(1.48)	(1.93)
Physical condition	0.6060^***^	−0.0023	−0.0124^***^	−0.0993^***^	0.0184^**^	0.0957^***^
	(4.47)	(−1.33)	(−2.72)	(−4.80)	(2.35)	(4.39)
Sleep quality	0.3200^***^	−0.0012	−0.0066^**^	−0.0525^***^	0.0097^*^	0.0506^***^
	(2.70)	(−1.27)	(−2.13)	(−2.74)	(1.88)	(2.69)
Academic performance	0.3730^**^	−0.0014	−0.0076^*^	−0.0611^**^	0.0113	0.0589^**^
	(2.07)	(−1.19)	(−1.79)	(−2.07)	(1.58)	(2.07)
*N*	515					

t-statistics in parentheses: ^*^p < 0.1, ^**^p < 0.05, ^***^p < 0.01.

#### 3.2.2. Different types of companionship and parental roles in relation to children's well-being

Table 5 shows that the average time that parents spent with their children in life and leisure was 0.6385 h, and the average time in educational interaction was 0.4124 h. Mothers spent an average of 0.4718 h with their children on activities related to life and leisure, and they spent 0.3139 h on educational interactions. The corresponding values for fathers were 0.1666 h on life and leisure activities and 0.0984 h on educational interactions. See Table 5 for details.

**Table 5:** Descriptive statistics for different types of time spend with children and different parental roles.

**Variable**		**Mean**	**SD**	**Min**	**Max**
Total time	Life and leisure	0.6385	2.1045	0	17.7500
	Educational interaction	0.4124	1.2064	0	9.4667
Mothers' time spent with children	Life and leisure	0.4718	1.7318	0	12.8333
	Educational interaction	0.3139	1.0327	0	9.4667
Fathers' time spent with children	Life and leisure	0.1666	0.8671	0	9.9500
	Educational interaction	0.0984	0.4836	0	5.4833

This study further examines the effect of the different roles of parents on children's well-being, as shown in Table 6. Model 1 was the regression of the total time that parents spent on life and leisure and education interactions in relation to children's well-being. The results showed that the more time they spent on life and leisure activities with children, the higher the children's well-being was, but the relationship between educational interaction time and children's well-being was not significant. Model 2 was the regression of the time mothers spent with children on life and leisure activities and educational interactions in relation to children's well-being. The results showed a positive correlation between life and leisure time and well-being, but the relationship with educational interaction was not significant. Model 3 was the regression of the time fathers spent on life and leisure and education interactions in relation to children's well-being. The results demonstrated that children's well-being improved when fathers spent time with their children in life and leisure activities and educational interactions. See Table 6 for details.

**Table 6:** Regression results of different types of companionship and parental division of labor on children's well-being.

	**Type of companionship**	**Model1**	**Model2**	**Model3**
Total time	Life and leisure	0.1020^***^		
		(2.59)		
Educational interaction	0.1030			
	(1.35)			
Mothers' time spent with children	Life and leisure		0.1030^**^	
			(2.07)	
Educational interaction		0.0611	
		(0.76)	
Fathers' time spent with children	Life and leisure			0.1790^**^
			(2.17)
Educational interaction			0.3630^*^
			(1.88)
Gender		−0.1520	−0.1620	−0.1660
		(−0.87)	(−0.92)	(−0.96)
Age		−0.6920^**^	−0.7380^***^	−0.6670^**^
		(−2.45)	(−2.61)	(−2.36)
Age^2^		0.0302^**^	0.0318^***^	0.0289^***^
		(2.52)	(2.66)	(2.41)
Nation		0.1730	0.1400	0.1630
		(0.62)	(0.50)	(0.59)
Areas		0.3620	0.3310	0.4390^*^
		(1.53)	(1.41)	(1.85)
Family income		0.0895^*^	0.0876^*^	0.0987^**^
		(1.91)	(1.87)	(2.08)
Physical condition		0.6060^***^	0.6020^***^	0.6010^***^
		(4.47)	(4.46)	(4.44)
Sleep quality		0.3210^***^	0.3210^***^	0.3050^***^
		(2.68)	(2.70)	(2.61)
Academic performance		0.3730^**^	0.3640^**^	0.3920^**^
		(2.07)	(2.02)	(2.19)
*N*		515		

t-statistics in parentheses: ^*^p < 0.1, ^**^p < 0.05, ^***^p < 0.01.

### 3.3. Heterogeneity analysis

Children's well-being hinges on their perceived ability to do well in school (Chang et al., 2015). Therefore, the learning environment may be an important factor that affected their perception of how much time parents spend with them. Our study analyzed the differences stemming from children's different learning situations from the perspectives of stages of learning and academic performance.

Models 4 and 5 divided the total sample into primary school and middle school, and the numbers of children in each model were 289 and 226, respectively. The results showed that greater amounts of time spent with children had a positive effect on the well-being of primary school and middle school children.

Models 6 and 7 were divided into samples of children with worse and better academic performance, and the numbers were 220 and 295, respectively. The results showed that, for children with the worst academic performance, the time parents spent with their children did not improve their well-being and that better academic performing children preferred spending time with parents. See Table 7 for details.

**Table 7:** Heterogeneity regression results of time spent with children on children's well-being.

	**Model 4**	**Model 5**	**Model 6**	**Model 7**
	**Primary school children**	**Middle school children**	**Worse academic performance**	**Better academic performance**
Parents' time spent with children	0.0989^**^	0.1240^*^	0.0846	0.1140^**^
	(2.35)	(1.72)	(1.54)	(2.37)
Gender	−0.2740	−0.0800	0.1320	−0.4240^*^
	(−1.12)	(−0.31)	(0.48)	(−1.80)
Age	0.7200	0.4660	−0.5760	−1.043^***^
	(0.81)	(0.12)	(−1.32)	(−2.59)
Age^2^	−0.0434	−0.0034	0.0219	0.0482^***^
	(−0.93)	(−0.03)	(1.21)	(2.79)
Nation	−0.0550	0.3090	0.5690	−0.1390
	(−0.16)	(0.59)	(1.59)	(−0.35)
Areas	0.2860	0.6270^**^	0.3470	0.3850
	(0.72)	(2.00)	(1.11)	(1.06)
Family income	0.0173	0.1970^**^	0.0790	0.0947
	(0.35)	(2.50)	(1.14)	(1.50)
Physical condition	0.5830^***^	0.5900^***^	0.6650^***^	0.5340^***^
	(3.21)	(2.75)	(3.37)	(2.75)
Sleep quality	0.4610^***^	0.1240	0.4690^***^	0.1350
	(2.99)	(0.62)	(2.64)	(0.84)
Academic performance	0.0907	0.8070^***^		
	(0.38)	(2.74)		
*N*	289	226	220	295

t-statistics in parentheses: ^*^p < 0.1, ^**^p < 0.05, ^***^p < 0.01.

## 4. Discussion

Our study aimed to discover the effect of the time parents spend with children on children's well-being in China. Different from previous studies, we used time to measure parental companionship. The results showed that the time parents spent with children could improve children's well-being. Fathers spent less time with children than mothers, both in terms of time spent on life and leisure and educational interactive time. Fathers' time spent in educational interactions had a significant effect on children's well-being. Further subsample regressions found that children's academic performance was a significant factor influencing the impact of the time parents spent with children on children's well-being.

Our findings suggested that the more time parents spent with their children, the higher their well-being. This was consistent with previous studies (Mínguez, 2019). This may be due to the fact that children needed emotional support from their parents and that parents had an important influence on the emotional and psychological health of their children (Güngör et al., 2019). The more time parents spent with children, the more family warmth the children experienced. Parents transmitted positive or negative emotions to their children in their interactions (Fischer et al., 2021). The way and type of time parents spent with their children influenced children's well-being (Fallesen and Ghler, 2019). However, there were studies that suggested that improving the quality of companionship could facilitate parent–child relationships (Roeters et al., 2010). What may be important for children was not only the amount of time spent with them but also the quality of companionship.

On the type of parental companionship, consistent with previous studies (Renk et al., 2003), parents spent more time with their children's life and leisure than they did in educational interactive time. A further division of parental roles found that mothers spent more time with their children than fathers, both in life and leisure time and educational interactive time. Previous studies have shown that, despite long work hours, fathers could still make time for their children (Coles et al., 2017), and the father's companionship time increased more than the mother's (Cano, 2019). However, in most cases, mothers spent too much of their free time supervising their children, while fathers engaged in non-child-related activities (Pablo and Joan, 2018) and mothers provided the majority of childcare services (Lawrence et al., 2019). The greater the father's educational interaction time, the higher the children's well-being, whereas the mother's educational interaction time had no significant effect on children's well-being. This may be because fathers may have more choices in the educational interactions they did with their children (Renk et al., 2003). Fathers chose activities that were more meaningful to them and less energy-demanding (Roeters and Gracia, 2016). Fathers' time spent on educational interactions had a positive impact on all types of parenting (Kalenkoski and Foster, 2008). Fathers would choose the more enjoyable aspects of companionship, and mothers may make higher demands from children (Craig, 2006). Thus, the more time spent in educational interactions, the children did not necessarily have a higher sense of happiness. Time spent on educational interactions was more likely to increase children' well-being than time spent on life and leisure (Thomsen, 2015; Roeters and Gracia, 2016), and attention should be paid to the type of educational interaction.

Our findings showed that, the more time parents spent with their children, whether primary or middle school children, the higher the children's well-being. For children with worse academic performance, the time parents spent with children did not increase their well-being, and the more time parents spent with children, the higher the children's well-being with better academic performance. Chinese parents had high expectations for their children's education (Tong et al., 2020). Children with worse academic performance may tend to have some time to themselves, but they do not want to be without their parents for long periods (Lewis et al., 2008). This may also be because a neglectful educational style that lacks emotional communication and demands could hinder a children's academic development (Yang and Zhao, 2020). In this case, parents should pay regular attention to their needs and actively accompany them in their studies (Zheng and Hu, 2021).

## 5. Conclusion

This study was the first to find out the effect of parental companionship on children's well-being from the perspective of time use. “Don't let children lose at the starting line” has become the consensus of Chinese parents. Paying attention to the growth of children was the “number one task” for parents. However, there were some misunderstandings in carrying out this task, such as attaching importance to economic investment in the growth of children, lacking awareness of the effects of spending time with parents, and the effect of interaction with parents and children on children's well-being. According to the results of this study, children demonstrated positive emotions when parents spent time with them and increasing that time was the key to improving children's well-being. In particular, the types of time parents spent with children and the division of parental roles had different effects on children's well-being and children prefer life and leisure time and prefer to be with their fathers. Some fathers did take on co-parenting, sharing the various tasks equally with mothers, while others conformed to the “absent father” stereotype and participated very little in the activities covered in our questionnaire (Monteiro et al., 2009). In this sense, fathers should find more time to be with their children in activities to promote children's well-being. In addition, children of different groups were affected differently by the time parents spent with their children, and more attention needs to be devoted to the differing needs of children. The time spent with children could be improved in the following ways. It was necessary to provide family guidance and education services, explore the implementation of “Home office” and other public policies, and coordinate family education services and children's mental health services. The time spent with children should be improved. “Everything has to do with children” (Bouma et al., 2019) was the theme of family activities. In particular, fathers should take on more family companionship functions. We should also pay attention to the individual needs of children and demonstrate more empathy for them. Children had their own independent thinking needs (López-Pérez and Fernández-Castilla, 2017). Parents should pursue an optimal division of labor in the family and exert less control but participate more in children's growth (Garcia Mendoza et al., 2019), providing more effective empathy and companionship. Therefore, future studies should focus more on the effect of the quality of parental companionship on children's well-being and whether this effect varies by parental role and type of companionship.

## 6. Limitations

An important limitation of the study was that the quality of the parent–child relationship was not taken into account. When a parent spends a given number of hours with their child, the parent may be hitting the children, yelling at them, giving them a hug, listening to a child's problem, and supporting them, among others. It would be reductionist to consider only the amount of time parents and children spend together and forget the quality of that relationship. The quality, rather than the quantity of time spent with the child, has an impact on the child's adjustment. Lower quality and more time for companionship may not improve children's well-being. Higher-quality companionship included more than just the time parents spent with their children, and it also included the emotional component of time (Vaterlaus et al., 2019). More attention to children and improved quality of companionship could foster parent–child relationships (Roeters et al., 2010). Another limitation of the study is that cross-sectional data do not allow predictions to be made about the impact of the time parents spend with their children on their well-being or to assess changes in parental time use across developmental stages, which should be argued. In addition, owing to limitations of data availability, we were neither able to provide more information on the socioeconomic level of each family nor able to investigate any differences in the function of school type (primary vs. middle school) and academic performance (worse vs. better) on all study variables.

## Data availability statement

The original contributions presented in the study are included in the article/supplementary material, further inquiries can be directed to the corresponding author.

## Author contributions

DL: conception, design, data analysis and interpretation, writing the original draft, and supervision. XG: conception, design, funding acquisition, and supervision. All authors contributed to the article and approved the submitted version.

## References

[B1] Acar I. H. Ucus S. Yildiz S. (2017). Parenting and Turkish children's behaviour problems: the moderating role of qualities of parent–child relationship. Early Child Dev. Care 14, 1–14. 10.1080/03004430.2017.13653

[B2] Baumrind D. (1968). Authoritarian vs. authoritative parental control. Adolescence 3, 255–272.

[B3] Baumrind D. (1971). Current patterns of parental authority. Dev. Psychol. 4, 1–103. 10.1037/h0030372

[B4] Baumrind D. (1991a). “Effective parenting during the early adolescent transition,” in Advances in Family Research Series: Family Transitions, eds P. A. Cowan, and E. M. Herington (Mahwah: Lawrence Erlbaum Associates, Inc.), 111–163.

[B5] Baumrind D. (1991b). “Parenting styles and adolescent development,” in Encyclopedia of Adolescence, eds R. M. Lerner, A. C. Petersen, and J. Brooks-Gunn (Garland: Lawrence Erlbaum Associates, Inc), 746–758.

[B6] Bonke J. Greve J. (2012). Children's health-related life-styles: how parental child care affects them. Rev. Econ. Household 10, 557–572. 10.1007/s11150-012-9157-6

[B7] Bouma H. Grietens H. López López M. Knorth E. J. (2019). Learning from parents: a qualitative interview study on how parents experience their journey through the Dutch child protection system. Child Fam. Soc. Work 25, 116–125. 10.1111/cfs.12723

[B8] Cano T. (2019). Changes in fathers' and mothers' time with children: Spain, 2002–2010. Eur. Sociol. Rev. 35, 616–636. 10.1093/esr/jcz020

[B9] Carvalho J. M. S. Delgado P. Montserrat C. Llosada-Gistau J. Casas F. (2021). Subjective wellbeing of children in care: comparison between Portugal and Catalonia. Child Adolesc. Soc. Work J. 38, 81–90. 10.1007/s10560-020-00675-3

[B10] Cha S.-E. Song Y.-J. (2016). Time or money: the relationship between educational attainment, income contribution, and time with children among Korean fathers. Soc. Indicat. Res. 134, 195–218. 10.1007/s11205-016-1414-2

[B11] Chang L. Mcbride-Chang C. Stewart S. Au E. (2015). Life satisfaction, self-concept, and family relations in Chinese adolescents and children. Int. J. Behav. Dev. 27, 182–189. 10.1080/01650250244000182

[B12] Chao R. K. (2001). Extending research on the consequences of parenting style for Chinese Americans and European Americans. Child Dev. 72, 1832–1843. 10.1111/1467-8624.00381 11768148

[B13] Chen W. W. Wu C. W. Yeh K. H. (2016). How parenting and filial piety influence happiness, parent–child relationships and quality of family life in Taiwanese adult children. J. Fam. Stud. 22, 80–96. 10.1080/13229400.2015.1027154

[B14] Chi P. Du H. King R. B. Zhou N. Cao H. Lin X. (2019). Wellbeing contagion in the family: transmission of happiness and distress between parents and children. Child Indicat. Res. 12, 2189–2202. 10.1007/s12187-019-09636-4

[B15] Climent-Galarza S. Alcaide M. Garcia O. F. Chen F. Garcia F. (2022). Parental socialization, delinquency during adolescence and adjustment in adolescents and adult children. Behav. Sci. 12, 448. 10.3390/bs12110448 36421744 PMC9687913

[B16] Coles L. Hewitt B. Martin B. (2017). Contemporary fatherhood: social, demographic and attitudinal factors associated with involved fathering and long work hours. J. Sociol. 14, 144078331773969. 10.1177/1440783317739695

[B17] Craig L. (2006). Does father care mean fathers share? A comparison of how mothers and fathers in intact families spend time with children. Gend. Soc. 20, 259–281. 10.1177/0891243205285212

[B18] Darling N. Steinberg L. (1993). Parenting style as context: an integrative model. Psychol. Bull. 113, 487–496. 10.1037/0033-2909.113.3.487

[B19] Deater-Deckard K. Dodge K. A. Bates J. E. Pettit G. S. (1996). Physical discipline among African American and European American mothers: links to children's externalizing behaviors. Dev. Psychol. 32, 1065–1072. 10.1037/0012-1649.32.6.1065

[B20] Du F. L. Su J. C. Yang X. S. (2020). Division of housework and family happiness. Stud. Labor Econ. 8, 64–86.

[B21] Du F. L. Wang W. W. Dong X. Y. (2019). Where Does the Time Go? China Time Use Survey Research. Beijing: China Social Sciences Press.

[B22] Dwairy M. Achoui M. (2006). Introduction to three cross-regional research studies on parenting styles, individuation, and mental health in Arab societies. J. Cross-Cult. Psychol. 37, 221–229. 10.1177/0022022106286921

[B23] Fallesen P. Ghler M. (2019). Family type and parents time with children: longitudinal evidence for Denmark. Acta Sociol. 63, 000169931986852. 10.1177/0001699319868522

[B24] Fan H. Li D. Zhou W. Jiao L. Zhang L. (2020). Parents' personality traits and children's subjective wellbeing: a chain mediating model. Curr. Psychol. 19, 1–12. 10.1007/s12144-020-01078-4

[B25] Fischer I. Schober P. S. Nagengast B. (2021). Parental relationship quality and children's behavioral problems: childcare quality as a protective factor? J. Fam. Res. 33, 703–733. 10.20377/jfr-379

[B26] Fuentes M. C. Garcia O. F. Alcaide M. Garcia-Ros R. Garcia F. (2022). Analyzing when parental warmth but without parental strictness leads to more adolescent empathy and self-concept: evidence from Spanish homes. Front. Psychol. 13, 821. 10.3389/fpsyg.2022.1060821 36544447 PMC9760939

[B27] Gahramanov E. Hasanov R. Tang X. (2019). Parental involvement and Children's human capital: a tax-subsidy experiment. Econ. Model. 85, 16–29. 10.1016/j.econmod.2019.05.002

[B28] Garcia Mendoza M. D. C. Sanchez Queija I. Parra Jimenez A. (2019). The role of parents in emerging Adults' psychological wellbeing: a person-oriented approach. Fam. Process 58, 954–971. 10.1111/famp.12388 30198562

[B29] Garcia F. Gracia E. (2009). Is always authoritative the optimum parenting style*?* Evidence from Spanish families. Adolescence 44, 101–131. 19435170

[B30] Garcia F. Gracia E. (2014). “The indulgent parenting style and developmental outcomes in South European and Latin American countries,” in Parenting Across Cultures, ed H. Selin (New York, NY: Springer), 419–433. 10.1007/978-94-007-7503-9_31

[B31] Garcia O. F. Fuentes M. C. Gracia E. Serra E. Garcia F. (2020). Parenting warmth and strictness across three generations: parenting styles and psychosocial adjustment. Int. J. Environ. Res. Public Health 17, 7487. 10.3390/ijerph17207487 33076230 PMC7602436

[B32] Garcia O. F. Serra E. (2019). Raising children with poor school performance: parenting styles and short- and long-term consequences for adolescent and adult development. Int. J. Environ. Res. Public Health 16, 1089. 10.3390/ijerph16071089 30934673 PMC6480465

[B33] Garcia O. F. Serra E. Zacares J. J. Garcia F. (2018). Parenting styles and short- and long-term socialization outcomes: a study among Spanish adolescents and older adults. Psychosoc. Intervent. 27, 153–161. 10.5093/pi2018a21

[B34] Gimenez-Serrano S. Alcaide M. Reyes M. Zacarés J. J. Celdrán M. (2022). Beyond parenting socialization years: the relationship between parenting dimensions and grandparenting functioning. Int. J. Environ. Res. Public Health 19, 4528. 10.3390/ijerph19084528 35457396 PMC9026310

[B35] Gimenez-Serrano S. Garcia F. Garcia O. F. (2021). Parenting styles and its relations with personal and social adjustment beyond adolescence: is the current evidence enough? Eur. J. Dev. Psychol. 19, 749–769. 10.1080/17405629.2021.1952863

[B36] Gugl E. Welling L. (2011). Time with sons and daughters. Rev. Econ. Household 10, 277–298. 10.1007/s11150-011-9129-2

[B37] Güngör H. Gülay Ogelman H. Körük,çü Ö. Erten Sarikaya H. (2019). The predictive effect of fathers' durations of working and spending time with their children on the aggression level of 5–6 year-old children. Early Child Dev. Care 430, 1–8. 10.1080/03004430.2019.1645137

[B38] Hagquist C. (2016). Family residency and psychosomatic problems among adolescents in Sweden: the impact of child-parent relations. Scand. J. Public Health 44, 36–46. 10.1177/1403494815610664 26487762

[B39] Jimenez T. I. Estevez E. Velilla C. M. Martin-Albo J. Martinez M. L. (2019). Family communication and verbal child-to-parent violence among adolescents: the mediating role of perceived stress. Int. J. Environ. Res. Public Health 16, 4538. 10.3390/ijerph16224538 31744062 PMC6888577

[B40] Kalenkoski C. M. Foster G. (2008). The quality of time spent with children in Australian households. Rev. Econ. Household 6, 243–266. 10.1007/s11150-008-9036-3

[B41] Lamborn S. D. Mounts N. S. Steinberg L. Dornbusch S. M. (1991). Patterns of competence and adjustment among adolescents from authoritative, authoritarian, indulgent, and neglectful families. Child Dev. 62, 1049–1065. 10.2307/1131151 1756655

[B42] Lawrence J. Haszard J. J. Taylor B. Galland B. Gray A. Sayers R.. (2019). A longitudinal study of parental discipline up to 5 years. J. Fam. Stud. 57, 1–18. 10.1080/13229400.2019.1665570

[B43] Lewis J. Noden P. Sarre S. (2008). Parents' working hours: adolescent children's views and experiences. Child. Soc. 22, 124. 10.1111/j.1099-0860.2007.00124.x

[B44] Liang B. Ji H. (2020). The effect of unemployment insurance on job search efforts: evidence from the Chinese time use survey. Econ. Res. 55, 179–197.

[B45] Liu G. Zhao Z. Li B. Pan Y. Cheng G. (2022). Parental psychological wellbeing and parental emotional warmth as mediators of the relationship between family socioeconomic status and children's life satisfaction. Curr. Psychol. 22, 1–8. 10.1007/s12144-022-03568-z

[B46] López-Pérez B. Fernández-Castilla B. (2017). Children's and adolescents' conceptions of happiness at school and its relation with their own happiness and their academic performance. J. Happiness Stud. 19, 1811–1830. 10.1007/s10902-017-9895-5

[B47] Maccoby E. E. Martin J. A. (1983). “Socialization in the context of the family: parent–child interaction,” in Handbook of Child Psychology, ed P. H. Mussen (Amsterdam: Wiley), 1–101.

[B48] Maftei A. Holman A.-C. Carlig E.-R. (2020). Does your child think you're happy? Exploring the associations between children's happiness and parenting styles. Child. Youth Serv. Rev. 115, 105074. 10.1016/j.childyouth.2020.105074

[B49] Martinez I. Garcia F. Veiga F. Garcia O. F. Rodrigues Y. Serra E. (2020). Parenting styles, internalization of values and self-esteem: a cross-cultural study in Spain, Portugal and Brazil. Int. J. Environ. Res. Public Health 17, 2370. 10.3390/ijerph17072370 32244451 PMC7177516

[B50] Martínez I. Murgui S. Garcia O. F. Garcia F. (2019). Parenting in the digital era: protective and risk parenting styles for traditional bullying and cyberbullying victimization. Comput. Hum. Behav. 90, 84–92. 10.1016/j.chb.2018.08.036

[B51] Martínez I. Murgui S. Garcia O. F. Garcia F. (2021). Parenting and adolescent adjustment: the mediational role of family self-esteem. J. Child Fam. Stud. 30, 1184–1197. 10.1007/s10826-021-01937-z

[B52] Martinez-Escudero J. A. Villarejo S. Garcia O. F. Garcia F. (2020). Parental socialization and its impact across the lifespan. Behav. Sci. 10, 101. 10.3390/bs10060101 32560214 PMC7349393

[B53] McAuley C. McKeown C. Merriman B. (2012). Spending time with family and friends: children's views on relationships and shared activities. Child Indicat. Res. 5, 449–467. 10.1007/s12187-012-9158-2

[B54] Milkie M. A. Kendig S. M. Nomaguchi K. M. Denny K. E. (2010). Time with children, children's wellbeing, and work-family balance among employed parents. J. Marriage Fam. 72, 1329–1343. 10.1111/j.1741-3737.2010.00768.x

[B55] Mínguez A. M. (2019). Children's relationships and happiness: the role of family, friends and the school in four European countries. J. Happiness Stud. 21, 1859–1878. 10.1007/s10902-019-00160-4

[B56] Monteiro L. Veríssimo M. Vaughn B. E. Santos A. J. Torres N. Fernandes M. (2009). The organization of children's secure base behaviour in two-parent Portuguese families and father's participation in child-related activities. Eur. J. Dev. Psychol. 7, 545–560. 10.1080/17405620902823855

[B57] Nie Q. Tian L. Huebner E. S. (2020). Relations among family dysfunction, loneliness and life satisfaction in Chinese children: a longitudinal mediation model. Child Indicat. Res. 13, 839–862. 10.1007/s12187-019-09650-6

[B58] Pablo G. Joan G. R. (2018). Child and adolescent developmental activities and time use in Spain: the gendered role of parents' work schedules and education levels. Eur. Sociol. Rev. 5, 518–538. 10.1093/esr/jcy029

[B59] Palacios I. Garcia O. F. Alcaide M. Garcia F. (2022). Positive parenting style and positive health beyond the authoritative: self, universalism values, and protection against emotional vulnerability from Spanish adolescents and adult children. Front. Psychol. 13, 6282. 10.3389/fpsyg.2022.1066282 36591008 PMC9800864

[B60] Park J. Jung H. J. Han Y. (2022). Latent profile analysis of associations among children's risk profiles, rights, and subjective wellbeing across 16 countries. Curr. Psychol. 16, 1–14. 10.1007/s12144-022-02916-3

[B61] Perez-Gramaje A. F. Garcia O. F. Reyes M. Serra E. Garcia F. (2020). Parenting styles and aggressive adolescents: relationships with self-esteem and personal maladjustment. Eur. J. Psychol. Appl. Legal Context 12, 1–10. 10.5093/ejpalc2020a1

[B62] Queiroz P. Garcia O. F. Garcia F. Zacares J. J. Camino C. (2020). Self and nature: parental socialization, self-esteem, and environmental values in Spanish adolescents. Int. J. Environ. Res. Public Health 17, 3732. 10.3390/ijerph17103732 32466198 PMC7277642

[B63] Rafferty K. A. Beck G. McGuire M. (2020). When facing hopeful and hopeless experiences: using snyder's hope theory to understand parents' caregiving experiences for their medically complex child. J. Pediatr. Health Care 34, 542–549. 10.1016/j.pedhc.2020.06.003 32771340

[B64] Rees G. Dinisman T. (2015). Comparing children's experiences and evaluations of their lives in 11 different countries. Child Indicat. Res. 8, 5–31. 10.1007/s12187-014-9291-1

[B65] Renk K. Roberts R. Roddenberry A. Luick M. Hillhouse S. Meehan C.. (2003). Mothers, fathers, gender role, and time parents spend with their children. Sex Roles 48, 305–315. 10.1023/A:102293441291012290119

[B66] Roeters A. Gracia P. (2016). Child care time, parents' wellbeing, and gender: evidence from the American time use survey. J. Child Fam. Stud. 25, 2469–2479. 10.1007/s10826-016-0416-7 27440990 PMC4933733

[B67] Roeters A. Lippe T. V. D. Kluwer E. S. (2010). Work characteristics and parent-child relationship quality: the mediating role of temporal involvement. J. Marriage Fam. 72, 1317–1328. 10.1111/j.1741-3737.2010.00767.x

[B68] Savahl S. Adams S. Florence M. Casas F. Mpilo M. Isobell D.. (2019). The relation between children's participation in daily activities, their engagement with family and friends, and subjective wellbeing. Child Indicat. Res. 13, 1283–1312. 10.1007/s12187-019-09699-333182491

[B69] Savahl S. Casas F. Adams S. (2021). The structure of children's subjective wellbeing. Front. Psychol. 12, 650691. 10.3389/fpsyg.2021.650691 34177705 PMC8225927

[B70] Steinberg L. (2005). “Psychological control: style or substance?,” in New Directions for Child and Adolescent Development: Changes in Parental Authority During Adolescence, ed J. G. Smetana (San Francisco: Jossey-Bass), 71–78. 10.1002/cd.129 16121898

[B71] Steinberg L. Lamborn S. D. Darling N. Mounts N. S. Dornbusch S. M. (1994). Over-Time changes in adjustment and competence among adolescents from authoritative, authoritarian, indulgent, and neglectful families. Child Dev. 65, 754–770. 10.2307/1131416 8045165

[B72] Steinberg L. Mounts N. S. Lamborn S. D. Dornbusch S. M. (1991). Authoritative parenting and adolescent adjustment across varied ecological niches. J. Res. Adolesc. 1, 19–36.

[B73] Thoilliez B. (2011). How to grow up happy: an exploratory study on the meaning of happiness from children's voices. Child Indicat. Res. 4, 323–351. 10.1007/s12187-011-9107-5

[B74] Thomsen M. K. (2015). Parental time investments in children. Acta Sociol. 58, 249–263. 10.1177/0001699315572159

[B75] Tong Y. Li J. X. Shu B. (2020). Is children's academic performance valuable to parents? Linking children's effort vs. results and fathers' vs. mothers' subjective wellbeing. Child Indicat. Res. 1, 763. 10.1007/s12187-020-09763-3

[B76] Vanassche S. Swicegood G. Matthijs K. (2012). Marriage and children as a key to happiness? Cross-national differences in the effects of marital status and children on wellbeing. J. Happiness Stud. 14, 501–524. 10.1007/s10902-012-9340-8

[B77] Vaterlaus J. M. Beckert T. E. Schmitt-Wilson S. (2019). Parent–child time together: the role of interactive technology with adolescent and young adult children. J. Fam. Issues 40, 2179–2202. 10.1177/0192513X19856644

[B78] Villarejo S. Martinez-Escudero J. A. Garcia O. F. (2020). Parenting styles and their contribution to children personal and social adjustment. Ansiedad y Estrés 26, 1–8. 10.1016/j.anyes.2019.12.001

[B79] Vujcic M. T. Brajsa-Zganec A. Franc R. (2019). Children and young peoples' views on wellbeing: a qualitative study. Child Indicat. Res. 12, 791–819. 10.1007/s12187-018-9559-y

[B80] Yang J. Zhao X. (2020). Parenting styles and children's academic performance: evidence from middle schools in China. Child. Youth Serv. Rev. 113, 17. 10.1016/j.childyouth.2020.105017

[B81] Zheng M. Hu S. (2021). Unequal childhoods in china: parental education and children's time use. J. Commun. Psychol. 2, 22710. 10.1002/JCOP.22710/v2/response1 34551135

